# Nuclear dynamics of singlet exciton fission in pentacene single crystals

**DOI:** 10.1126/sciadv.abg0869

**Published:** 2021-06-25

**Authors:** Hélène Seiler, Marcin Krynski, Daniela Zahn, Sebastian Hammer, Yoav William Windsor, Thomas Vasileiadis, Jens Pflaum, Ralph Ernstorfer, Mariana Rossi, Heinrich Schwoerer

**Affiliations:** 1 Fritz-Haber-Institut der Max-Planck-Gesellschaft, Berlin 14195, Germany.; 2 Julius-Maximilians-Universität, Experimentelle Physik VI, Am Hubland, 97074 Würzburg, Germany.; 3 Bayerisches Zentrum für Angewandte Energieforschung, Magdalene-Schoch-Straße 3, 97074 Würzburg, Germany.; 4 Max-Planck-Institut für Struktur und Dynamik der Materie, 22761 Hamburg, Germany.

## Abstract

Singlet exciton fission (SEF) is a key process for developing efficient optoelectronic devices. An aspect rarely probed directly, yet with tremendous impact on SEF properties, is the nuclear structure and dynamics involved in this process. Here, we directly observe the nuclear dynamics accompanying the SEF process in single crystal pentacene using femtosecond electron diffraction. The data reveal coherent atomic motions at 1 THz, incoherent motions, and an anisotropic lattice distortion representing the polaronic character of the triplet excitons. Combining molecular dynamics simulations, time-dependent density-functional theory, and experimental structure factor analysis, the coherent motions are identified as collective sliding motions of the pentacene molecules along their long axis. Such motions modify the excitonic coupling between adjacent molecules. Our findings reveal that long-range motions play a decisive part in the electronic decoupling of the electronically correlated triplet pairs and shed light on why SEF occurs on ultrafast time scales.

## Teaser

Coherent and incoherent long-range atomic motions accompany singlet exciton fission in pentacene.

## INTRODUCTION

Organic molecular semiconductors have unique optoelectronic properties, combining the intrinsic optical characteristics of the individual molecules with the long-range correlations enabled by intermolecular coupling. Among these properties, the ability of several organic semiconductors to undergo singlet exciton fission (SEF) has drawn tremendous fundamental and applied research interest over the past decades, summarized in several review articles (*1*–*4*). SEF is the process by which an electronically excited singlet exciton *S*_1_ spontaneously splits into two triplet states *T*_1_ + *T*_1_. It may occur if the excess energy Δ*E*, defined as the energy difference between the *S*_1_ state and twice the lowest triplet state, is positive or if a small negative Δ*E* can be compensated by thermal energy. Owing to the ability to generate two electron-hole pairs per absorbed photon, the process bears high relevance for optoelectronic applications.

Since the discovery of SEF in the late 60s by measurements of the magnetic dependence of fluorescence (*5*), much emphasis has been placed on revealing the intermediate states and reaction pathways of the SEF process using time-resolved methods. The current understanding of the process involves three steps (*1*, *6*)

S1⇌(1)(TT)1→(2)(T..T)1→(3)T1+T1 (1)

In this equation, step (1) describes the formation of the electronically and spin-correlated triplet pair, ^1^(*TT*). The intermediate state ^1^(*T*. . *T*), formed during step (2), represents spatially separated triplet states, which are electronically independent but spin-correlated. Step (3) finally leads to two independent triplet excitons. Experiments on SEF dynamics have been almost exclusively based on photon excitation–photon probe schemes, such as transient absorption (TA) (*7*–*9*), two-dimensional (2D) spectroscopies (*10*, *11*), or photoelectron spectroscopies (*12*), all being electronic probes. In Eq. 1, step (2) confers most of their individual chemical and spectroscopic properties on the independent triplets; hence, it can be considered as the fission constituting process (*1*). Because of its implications for applications, measuring its characteristic time constant is of importance. While step (2) has been previously studied in pentacene dimers using TA spectroscopy, time scale extraction has remained challenging because of spectrally overlapping transitions (*6*).

SEF properties crucially depend on molecular structure and molecular packing in the crystal (*6*, *8*, *13*). Fission yields and rates vary vastly with orientations and distances of neighboring molecules, π-orbital correlations, Δ*E*, and sample purity. Furthermore, recent ultrafast spectroscopy studies in combination with theory indicate that SEF is intrinsically linked to nuclear motion (*10*, *14*–*18*). These studies call for an experimental probe that can access structural changes at the femtosecond time scale in a direct fashion. Time-resolved Raman spectroscopy may give some structural information on a molecular level but is limited if additional molecule-lattice coupling is involved (*15*, *19*). In contrast, ultrafast diffraction experiments with x-rays and electrons, established in the last decade, allow observation of structural changes on atomic time and length scales in crystalline materials with unit cell sizes up to several hundred atoms (*20*–*22*).

In this work, we directly reveal the structural dynamics accompanying the SEF process in single crystal pentacene using the method of femtosecond electron diffraction (FED), supported by real-time time-dependent density-functional theory (RT-TDDFT) and molecular dynamics simulations (MDS). Pentacene is the most widely studied SEF material and benefits from a wealth of previous theory and experimental works. Furthermore, the SEF process is exothermic at 300 K (Δ*E*≃ 100 meV) and occurs with a yield close to 100% (*2*, *23*). By resonantly exciting the lowest singlet exciton, excess heating of the crystal is avoided. This allows isolation of the structural dynamics arising from the SEF process. Our experiments reveal coherent and incoherent contributions to the structural dynamics, as well as a long-lived (≥1 ns), oriented structural distortion reflecting the polaronic character of the triplet excitons (*24*). The combination of RT-TDDFT, MDS, and diffraction analysis enables us to identify the coherent motion as a delocalized, collective sliding motion along the pentacene’s long axis. Furthermore, the mechanism of coherent phonon generation is assigned to exciton-phonon and subsequent phonon-phonon coupling. Our findings imply that both coherent and incoherent motions participate in the SEF process in pentacene on the picosecond time scale by enabling the electronic decoupling of ^1^(*TT*) into spatially separate triplets ^1^(*T*. . *T*).

## RESULTS

**Fig. 1:**
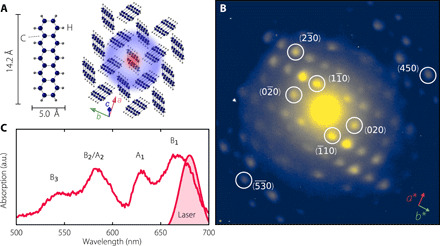
Overview of the equilibrium structural and optical properties of the pentacene crystal. (**A**) Illustrations of a single pentacene molecule (left) and of the crystal structure as viewed from a direction close to the *c* axis (long axis). The red-blue halo depicts charge separation and *S*_1_ exciton delocalization. The charge-transfer (CT) character of *S*_1_ in crystalline pentacene has been established by various theoretical (*43*, *61*–*65*) and experimental (*15*) approaches. Arrows indicate directions of crystal axes. (**B**) Exemplary transmission electron diffraction pattern from a 50-nm-thin (001) pentacene single crystal slab. Intensity in log scale, see linear scale representation in fig. S1. (**C**) Unpolarized linear absorption spectrum of the pentacene sample. B_1_, A_1_ and B_2_/A_2_, B_3_ are vibrational progressions of the lower and higher Davydov component at lower and higher energy, respectively, caused by a symmetric ring breathing mode of ≈170 meV, typical for polyacenes (*29*). The shaded red peak area reflects the optical pump spectrum resonant with the *S*_1_← *S*_0_ transition. a.u., arbitrary units.

Pentacene (C_22_H_14_) single crystals were grown by sublimation, giving rise to a triclinic crystal structure (space group *P*-1, number 2) (*25*, *26*). In contrast to nanocrystalline thin films of varying polymorphs and defect concentrations, the sublimation method yields controllable growth of large single crystals with a well-known crystal structure. The resulting herringbone structure, characteristic of polyacene crystals, is shown in Fig. 1A. Details about sample growth and crystal structure are given in Materials and Methods. An exemplary transmission electron diffraction pattern is shown in Fig. 1B, with the incident electron beam aligned normal to the crystal’s *ab* plane. Pentacene being a pure hydrocarbon, the overall diffraction intensity is low compared to metal-organic compounds or organic-inorganic charge-transfer (CT) complexes previously studied with FED (*21*, *22*, *27*, *28*). A diffuse scattering cloud is observed in the background, which we attribute to defects in the crystal and inelastic scattering.

### FED experiments

For the FED measurements, we use a 50-fs optical pump pulse with a central wavelength of 680 nm to resonantly excite the *S*_1_← *S*_0_ transition (B_1_) and populate the lower Davydov component (see Fig. 1C). The polarization of the pump pulse is tuned along the *a* axis of the crystal, according to the direction of the transition dipole moment of *S*_1_← *S*_0_ (the lower Davydov component) (see also fig. S2) (*29*–*31*). We apply an incident fluence of 0.4 mJ/cm^2^, yielding a calculated excitation density of 1 of 30 molecules (text S1). The photoexcited singlet excitons subsequently undergo exothermic fission, and a femtosecond electron bunch probes the crystal lattice at a delay time *t* after excitation, generating a diffraction pattern. This sequence is repeated for different delays. Further details about the FED instrument are available in Materials and Methods and elsewhere (*32*).

**Fig. 2:**
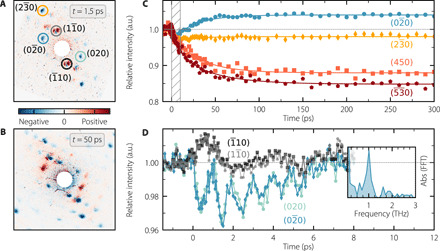
Structural dynamics accompanying the singlet exciton fission process in pentacene. (**A**) Difference between the diffraction pattern at *t* = 1.5 ps after photoexcitation and before photoexcitation. (**B**) Same as in (A) but at *t* = 50 ps. (**C**) Changes in intensity as a function of delay for selected Bragg reflections, indicated by circles in (A). Fits are obtained from a global fitting procedure performed on the ensemble of reflections (fig. S4). (**D**) Zoom into the first picoseconds for the (020), $\left(0\bar{2}0\right)$, $\left(\bar{1}10\right)$, and $\left(1\bar{1}0\right)$ reflections [(hatched gray area in (C)]. Inset shows the absolute value (Abs) of the FFT spectrum of the oscillating residuals for $\left(0\bar{2}0\right)$.

An overview of the photo-induced changes in the diffraction pattern is presented in Fig. 2 (A and B) in the form of intensity difference maps, obtained by subtracting the diffraction pattern before photoexcitation from the time-dependent patterns. Within our experimental resolution, no peak position shifts are observed. We thus treat size and shape of the unit cell as constant within the detected time window of 1 ns. However, we observe systematic intensity changes in various Bragg reflections, with certain intensities decreasing and others increasing following photoexcitation. Some orders, such as the (020) and $\left(1\bar{1}0\right)$, also switch sign as the dynamics proceed. Because thermal heating leads to an intensity decrease of all Bragg reflections, the observed intensity changes cannot be caused by heating effects only (see also the inelastic scattering signals around chosen Bragg reflections in fig. S3). In addition, we estimate the average excess energy per molecule to only around 3 meV, which corresponds to heating under equilibrium conditions of the crystal by 1 K (*33*). Therefore, beyond 50 ps, the observed intensity changes indicate a lattice distortion related to the electronic excitation (see text S2). The fact that the peak positions in reciprocal space do not change indicates that only a change in atomic positions within the unit cell occurs, which persists up to >1 ns.

Figure 2C shows time-dependent changes in intensity of few selected Bragg reflections. By performing a global fit to an ensemble of 16 time-dependent traces (see fig. S4), we show that the structural dynamics can be decomposed into a sum of two exponential contributions, a fast picosecond component of 1.6 ± 0.2 ps and a slower component with a time constant of 28 ± 1 ps. These time constants unambiguously reveal the time scales of the incoherent lattice dynamics accompanying the SEF process in pentacene.

To assign the observed time constants to physical processes, we consider previously reported complementary studies. Electronic spectroscopies have largely focused on step (1) of Eq. 1, which occurs within 100 fs in pentacene (*7*, *12*). This step is below our instrument response function of ≃250 fs, and we consider it instantaneous for our purposes. Step (3), the final breaking of the spin coherence between the triplet pair, was recently measured in time-resolved electron spin resonance experiments to be on the nanosecond time scale on tetracene, at least two orders of magnitude slower than the time constants observed here (*34*, *35*). Hence, we tentatively attribute these incoherent structural dynamics to the electronic decoupling of the electronically correlated triplet pair, (TT)1→(2)(T..T)1. We assign the fast time constant to adiabatic lattice reorganization following step (1) of Eq. 1 and the slower one to the formation of the spatially separated ^1^(*T*. . *T*) state via coupling to delocalized vibrational modes and lattice disorder (see discussion below).

In addition to the incoherent structural dynamics, a closer look into the first 10 ps reveals pronounced oscillations at 1 THz of the amplitudes of several Bragg reflections on top of the incoherent dynamics. This observation is consistent with a recent transient-absorption study on pentacene single crystals (*36*). Oscillations are found to be strongest along the b^*^ direction (indicated in Fig. 1B) as seen for the (020) and $\left(0\bar{2}0\right)$ reflections in Fig. 2D. While such oscillations are also present in several other diffraction orders (see fig. S5), we do not observe them in the a^*^ direction. A fast Fourier transform (FFT) of the oscillating parts of the Bragg reflections dynamics yields a peak at 1.0 ± 0.1 THz, shown in the inset of Fig. 2D. The decay time of the oscillations is 3.9 ± 0.6 ps and arises from energy dissipation and dephasing. To unravel the atomic motions behind the 1-THz oscillations in real space, we use an approach that combines RT-TDDFT with MDS. We show how this approach, which allows for a direct comparison with the FED experiments (*37*, *38*), is well suited given the complex vibrational structure of the pentacene crystal, featuring 108 phonon branches.

### Theoretical modeling: Nuclear dynamics

To complement the FED data and investigate the complex structural dynamics of the pentacene crystal in real space, we perform two types of simulations, namely, short ab initio Ehrenfest dynamics including a laser excitation as modeled by RT-TDDFT and long MDS in and out of equilibrium (using a hotspot thermostat centered at 1 THz) with an empirical force field. These simulations include the crystal periodicity and do not rely on the harmonic approximation for the nuclear motion. Details about these simulations are given in Materials and Methods.

**Fig. 3:**
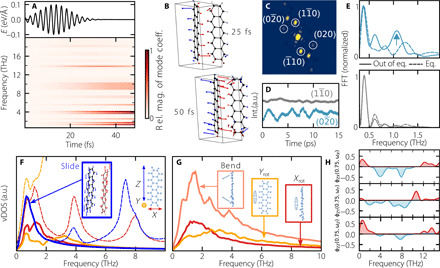
RT-TDDFT combined with MD simulations yield insights into the collective motions accompanying singlet fission in real space. (**A**) Projection of atomic displacements on the vibrational normal modes of the crystal, during Ehrenfest dynamics with a laser excitation as shown in the top panel (RT-TDDFT). Color code indicates the relative magnitudes, at each time step, of each normal mode’s coefficient. The harmonic frequencies of each mode are shown on the *y* axis. (**B**) Real-space atomic displacements of the crystal after 25 and 50 fs of Ehrenfest dynamics with respect to the structure at *t* = 0. Blue indicates that the *x* axis projection of the displacement has negative values and red otherwise. (**C**) Simulated diffraction pattern from classical MDS. (**D**) Intensity fluctuations of simulated diffraction peaks from equilibrium MDS corresponding to peaks shown in Fig. 2D. $\left(0\bar{2}0\right)$: blue, $\left(1\bar{1}0\right)$: gray. (**E**) FFT of the Bragg peak intensities shown in (D), matching colors. Dashed line: equilibrium MDS. Solid line: out-of-equilibrium MDS. Data have been normalized. (**F**) Partial equilibrium vDOS calculated for projections onto the *X*, *Y*, and *Z* axes (inset). Dashed lines represent data obtained for carbon atoms only; solid lines are for the molecular CM. (**G**) Partial vDOS patterns calculated for rotations of molecules around the *Y* and *X* axes and bending of the molecules (see the main text). (**H**) Selected slices (see the main text) from the 2D-vDOS auto- and cross-correlation patterns shown in fig. S9 revealing phonon-phonon coupling between different directions.

The RT-TDDFT simulations are performed in a single pentacene crystal unit cell of the polymorph that matches the experimental diffraction pattern. These short time dynamics probe the electron-phonon coupling in the initial steps of the singlet excitation. They show that specific phonon modes, primarily in the region below ≃15 THz, are activated following a laser excitation at 1.8 eV polarized along the *a* axis, shown in Fig. 1A. This is illustrated in Fig. 3 (A and B). Above 15 THz, we do not observe substantial activation. The results in Fig. 3A indicate that two groups of normal modes are predominantly activated by the laser: around 1 and 4 THz. In Fig. 3B, we show the atomic displacements observed after 25 and 50 fs of RT-TDDFT calculations. It is visible that the direction of these displacements is similar to the ones discussed in (*18*) for the singlet state, including a component in the direction of the mode at 5.4 THz (intermolecular rocking motion). We find, however, that the atomic motion accompanying the singlet excitation in the anharmonic potential energy surface (PES) of the crystal is best described by a combination of several modes. All activated modes and a quantitative analysis of the coefficients at each snapshot are shown in fig. S6 (see also text S3).

Further insight into the character of the crystal dynamics in more realistic systems is obtained from the results of MDS in and out of equilibrium within a 3 ×3×2 pentacene crystal cell of the same polymorph. Out-of-equilibrium MDS were obtained from microcanonical dynamics following a thermalization by a hotspot thermostat (*39*) centered at 1 THz. A connection to the FED experiments can be obtained by simulating diffraction patterns for each MDS snapshot (*37*, *40*), as illustrated in Fig. 3C. Figure 3D displays simulated Bragg peak intensities as a function of time in thermodynamic equilibrium for the $\left(0\bar{2}0\right)$ and $\left(1\bar{1}0\right)$ reflections. The $\left(0\bar{2}0\right)$ reflection fluctuates with a larger amplitude than the $\left(1\bar{1}0\right)$ reflection and with a period of roughly 2 ps. We stress that these are thermal fluctuations and coherent phonon excitations would have a larger amplitude. Figure 3E shows the FFT of these intensities, in equilibrium (dashed lines) and out of equilibrium obtained from the incoherent thermal excitation (solid lines). The $\left(0\bar{2}0\right)$ and $\left(1\bar{1}0\right)$ Bragg reflections are marked with blue and gray colors, respectively. While there is no clear 1-THz component in the $\left(0\bar{2}0\right)$ Bragg reflection obtained from the equilibrium MDS, this component clearly appears in the transient relaxation regime after vibrational excitation with the 1-THz hotspot thermostat. In contrast, the FFT of the $\left(1\bar{1}0\right)$ reflection is only slightly affected by the vibrational excitation with the 1-THz hotspot thermostat. No 1-THz component is observed in this Bragg reflection, either in equilibrium or in out-of-equilibrium MDS.

The characteristic motions around 1 THz deserve an in-depth analysis. Because of the complexity of the collective motions observed in the MDS of the large crystal supercell (see movie S1 and its description in the Supplementary Materials), we present a decomposition of these motions into specific components, namely, rigid-body motions of the molecules in the crystal and low-frequency intramolecular motions. This is achieved by calculating partial vibrational density of states (vDOS) from the Fourier transform of the velocity auto-correlation functions. In Fig. 3F, we show the partial vDOS of simulations in thermal equilibrium (300 K) of the selected coordinates defined by the three orthogonal axes of each individual molecule, shown in the inset of Fig. 3F: the direction parallel to the short edge of the molecule (*X*), the normal vector to the benzene rings of the molecule (*Y*), and the vector parallel to the long edge of the molecule (*Z*). Further details about the projection are provided in text S4. Specifically, Fig. 3F shows the projected vDOS of carbon atoms along *X*, *Y*, and *Z* (dashed lines), as well as the vDOS of the center of mass (CM) along *X*_CM_, *Y*_CM_, and *Z*_CM_ (solid lines).

These partial vDOS provide a clearer picture of the dynamical landscape. In particular, the *Z* projection (dashed blue) is characterized by two sharp peaks around 1 and 8 THz. The 1-THz peak along this coordinate can be fully accounted by CM motions along *Z* (solid blue line), as the dashed and solid blue lines overlap. This displacement of *Z*_CM_ involves entire molecules sliding along each other in a highly collective, intermolecular motion, which involves groups of molecules forming a wave along *Z* (see fig. S7, movie S2, and its description in the Supplementary Materials).

The MDS results also show that a realistic picture of atomic motion of crystals in the THz region cannot be reduced to one single component. Below 2 THz, the vDOS exhibits components of CM motions not only along *Z*, as evidenced in Fig. 3F. In addition, the projection of the atomic motions on the *X*, *Y*, and *Z* axes only present a partial picture of the complex dynamical features of this crystal. Further insights are obtained by tracking the CM motion of individual benzene rings in addition to the CM of entire molecules. This approach enables us to identify two rotations of the molecule around the *X* and *Y* directions, *X*_rot_ and *Y*_rot_, as well as an intramolecular bending motion of the molecule, *Z*_bend_. The rotation around *X* is related to the low-frequency mode that shows an activation in the RT-TDDFT simulations (see Fig. 3B). These motions, which all have a 1-THz component, are shown in Fig. 3G. Together, they lead to collective intra- and intermolecular motions of the molecules without altering the molecular CMs (see fig. S8, movie S3, and its description in the Supplementary Materials). In reality, the character of the vibrational modes that populate the 1-THz region at thermal equilibrium contains a combination of these motions, as expected in an anharmonic potential.

Last, we find pronounced phonon-phonon coupling of the low-THz region with higher-frequency phonon modes and between modes in different directions. 2D-vDOS correlation plots (*41*) of the motion along *X*, *Y*, and *Z* coordinates were analyzed, similarly to (*42*), where this method was also applied to molecular crystals. These plots are shown in fig. S9, and selected cuts ϕ_αβ_(ω_α_, ω_β_), α, β = *X*, *Y*, *Z*, are shown in Fig. 3H. The red (blue) color indicates positive (negative) correlation. These particular slices make it evident that (i) the low-frequency (0.5 to 1.5 THz) sliding motions along *Z*, shown in the inset of Fig. 3F, couple to motions around 4 THz and predominantly 8 THz in the same direction (the 8-THz motions are vertical pulsing of the molecules; see movie S4 and its description in the Supplementary Materials); (ii) these same sliding motions along *Z* strongly couple to motions along *X* around 1, 4, and 8 THz; and (iii) they also couple to the motions along *Y* in the regions between 2 to 4 THz and 6 to 8 THz. The motions along *Y* and *X*, to which the sliding *Z* motion couples, overlap with the ones populated upon the singlet excitation in the RT-TDDFT simulations. These results highlight the importance of phonon-phonon coupling in general for a realistic description of atomic motion in soft crystals.

## DISCUSSION

**Fig. 4:**
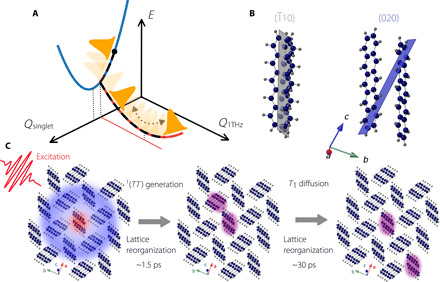
Schematic illustrations of coherent and incoherent lattice dynamics of singlet fission in pentacene. (**A**) Proposed mechanism of coherent phonon generation. The cartoon shows the excited PES on an energy scale *E* and wavepacket motion (orange) on this surface. The laser excites inter- and intramolecular modes (in particular around 4 THz) along the *Q*_singlet_ coordinate. These modes subsequently couple strongly to the 1-THz sliding motion via phonon-phonon coupling, initiating coherent wave packet dynamics along the 1-THz coordinate *Q*_1THz_. (**B**) The pentacene molecules cut through the (020) and the $\left(\bar{1}10\right)$ planes differently. The 1-THz sliding motions appears much more pronounced in the (020) plane compared to the $\left(\bar{1}10\right)$ plane. (**C**) Possible real-space pictures associated with the observed incoherent structural dynamics. The purple halos represent triplet excitons.

The combination of experiments and simulations yields previously unknown insights into the mechanisms of SEF in pentacene. By virtue of its strong CT character, photoexcitation of the *S*_1_ exciton generates a local charge separation, with a partially positive central pentacene molecule, surrounded by four partially negative molecules (*43*). The consequence of this charge separation is the instantaneous onset of a Coulomb force acting on the atoms of the crystal, which modifies their equilibrium position. Such a force explains the generation of coherent oscillations in the pentacene crystal via the displacive excitation mechanism (*44*), illustrated in Fig. 4A. Simulations reveal the microscopic mechanism of phonon generation: The RT-TDDFT simulations show that, at short times, the laser excitation results in the population of inter- and intramolecular modes in the crystal along the *a* and *b* axes, especially around 4 THz. The MDS reveal strong phonon-phonon coupling between these motions and the vertical sliding motion at 1 THz, close to the crystal’s *c* axis. In this picture, the experimentally observed coherent 1-THz motions thus arise from exciton-phonon and subsequent phonon-phonon coupling.

The consistency of the experimental signals with the sliding motion can be assessed using structure factor considerations. Modulations of a Bragg reflection intensity *I_hkl_* arise from modulation of the respective structure factor $F_{\mathrm{hkl}}=\sum_{j}f_{j}\cdot e^{i{\vec{G}}_{\mathrm{hkl}}\cdot \vec{r_{j}}}$, where *j* is the *j*th atom of the unit cell, $\vec{r_{j}}$ is its position, *f_j_* is the atomic form factor, and ${\vec{G}}_{\mathrm{hkl}}$ is a Bragg scattering vector for Miller indices (*hkl*). Molecular motion perpendicular to a lattice plane (*hkl*) changes the respective scattering intensity *I_hkl_*. In contrast, in-plane motions do not. We find that the 1-THz signal is observed in Bragg reflections for which the sliding motion has a perpendicular component to the lattice plane. No THz modulation is observed in Bragg reflections for which the sliding motion occurs within the lattice plane. Figure 4B shows two examples: the (020) plane, with a pronounced perpendicular component to the long axis of both inequivalent pentacene molecules and a pronounced 1-THz oscillation in the corresponding Bragg orders, and the $\left(\bar{1}10\right)$ plane, parallel to the molecules’ long axis, with a lack of 1-THz oscillation in the corresponding Bragg orders. All other investigated planes are shown in fig. S5. The experimental structural dynamics are thus consistent with the long-axis sliding motion. However, because of the vDOS of molecular crystals being rich of coupled molecular and lattice modes, a reduction of structure factor simulations onto individual molecular eigenmodes cannot reflect the full complexity of the structural dynamics.

Previous studies on excitonic coupling in pentacene have demonstrated that thermal lattice fluctuations of such low-frequency modes, and in particular of the sliding motion (*17*, *45*), cause substantial modulations of the electronic couplings between adjacent molecules, with direct influence on transport properties (*16*, *17*, *45*). These findings also apply to nonequilibrium situations, such as during the SEF process. The electronic decoupling of the ^1^(*TT*) state to the ^1^(*T*. . *T*) transient state, step (2) in Eq. 1, has been previously attributed to interactions with the phononic bath in the crystal (*1*). Our results allow us to suggest a mechanistic description of this process.

After photoexcitation, the formation of ^1^(*TT*) is accompanied by coherent atomic motions at 1 THz, as well as an initial incoherent lattice response with a time constant of 1.6 ps. This time constant is attributed to an adiabatic lattice reorganization adapting to the changes in the PES as a consequence of the ≈100-fs formation of the ^1^(*TT*) intermediate, step (1) in Eq. 1 (*7*, *12*). According to previous studies, the triplet-triplet exchange energy *J* determines the energy of the *^n^*(*TT*) states and stabilizes them below ^1^(*T*. . *T*) (*46*, *47*). The 1-THz motion modulates *J*, hence the splitting between the *^n^*(*TT*) states, and allows for admixture of higher *^n^*(*TT*) states such as ^5^(*TT*). As a result, the spatial separation of triplet wave functions increases, leading to a further decrease of *J*. Hence, in this scenario, the coherent 1-THz motion initiates and sustains the electronic decoupling of the ^1^(*TT*) state towards the spatially separated ^1^(*T*. . *T*) triplets. This process inherently modifies the PES again, leading to the second incoherent relaxation mechanism observed on the 28-ps time constant (see also Fig. 4C).

While being faster than spin dephasing (*34*, *35*), the slower time constant is indeed on the same order of magnitude as triplet exciton diffusion times in the *ab* plane of anthracene and tetracene single crystals (*48*, *49*) (see text S5). Considering the high exciton density in our measurements, this estimation indicates that the time constant attributed to lattice reorganization after ^1^(*TT*) separation is in a reasonable range for triplet hopping between adjacent molecules. We emphasize that the electronic mechanism and time scale of ^1^(*TT*) separation is not observed directly here, but its impact on the crystal lattice is, which follows the induced PES change.

Last, we observe the pronounced intensity increase for the (020) and $\left(0\bar{2}0\right)$ reflections on a time scale of tens of picoseconds while no changes in the structure factor are observed for the (*h*00) reflections. This indicates that the structural distortion effectively happens along the *b* axis of the crystal. Because this axis is the mean direction between nonequivalent pentacene neighbors (*b* + *a*/2 and *b − a*/2) and these nonequivalent pairs have the largest π-orbital overlap, one would expect attraction or repulsion caused by the long living triplet state interacting with its environment to induce exciton-polarons primarily in the *b* direction.

In summary, we have used FED to directly probe the photo-induced lattice dynamics in a prototypical SEF material. Our findings imply that both coherent and incoherent motions participate in the SEF process in pentacene on the picosecond time scale. The dominant atomic motions as well as the mechanism for coherent phonon generation have been identified by simulations and were found consistent with the experimental data. This study shows that including long-range, intermolecular structural dynamics is essential for an accurate description of the dynamics of SEF in pentacene, while considering motions within the unit cell only is not sufficient. We expect this finding to be relevant more generally for charge and energy transfer processes in molecular single crystals and polycrystalline thin films, as well as soft crystals like lead-halide perovskites.

## MATERIALS AND METHODS

### Pentacene crystal growth

Pentacene single crystals (C_22_H_14_) were grown via horizontal physical vapor deposition (*50*, *51*) using about 50 mg of twofold gradient sublimation purified pentacene. The starting material is placed in a horizontal furnace with a well-defined temperature gradient. The material is sublimed at 290^∘^C and transported by a continuous 30 sccm (standard cubic centimeters per minute) N_2_ (6 N purity) inert gas flow along the temperature gradient, leading to condensation of thin plate-like single crystals from supersaturated vapor in the colder zone of the furnace. The as-grown crystals were slowly cooled down over 12 hours to minimize thermal stress. By use of an ultramicrotome, crystals were subsequently cut parallel to their (*ab*) facets into platelets of hundreds of micrometers lateral size and 30 to 80 nm thickness to be used in FED experiments (see fig. S10). From out-of-plane x-ray diffraction measurements, the common pentacene bulk crystal phase, characterized by the (001) lattice distance of 14.1 Å, is identified (*25*, *26*, *52*). A comparison of the experimental electron diffraction pattern of the cut crystal platelets with simulated patterns is shown in fig. S11. This comparison allows us to determine the single crystal structure to be in the so-called high-temperature Siegrist phase, which is known to be metastable at room temperature (*26*). The diffraction pattern obtained by FED can be unambiguously assigned to this high-temperature phase for all investigated samples. We conclude that the microtome cutting does not change the crystalline integrity of the sample as clearly shown by the high-quality FED pattern.

### FED experiments

The output of a Ti:Sapph amplifier (Coherent, Astrella, 4 kHz, 6 W, 50 fs) is split into a pump and a probe path. The pump is derived from a commercial optical parametric amplifier (TOPAS; Light Conversion). For the probe, a home-built non-collinear optical parametric amplifier (NOPA) is used followed by a prism-compressor setup for dispersion compensation. The roughly 400-nJ NOPA pulses are focused onto a gold photocathode for the generation of femtosecond bunches of photoelectrons. The photoemitted electrons are accelerated toward the anode at 70 keV. More details about the electron gun design are provided in (*32*). We minimize the probe electrons’ propagation path to the sample, so as to minimize temporal broadening of the electron pulse due to space-charge effects. The electron bunch diffracts off the sample and a magnetic lens is used to focus the electrons onto a detector (F416; TVIPS). In this experiment, the temporal resolution is ≃250 fs. To analyze the Bragg reflection intensities quantitatively, each peak in the diffraction pattern is fitted with a 2D pseudo-Voigt function and a tilted background surface (see text S6 and fig. S12).

### Theoretical modeling

The TDDFT calculations performed in this project were carried out with the Octopus code (*53*, *54*) with the Perdew-Burke-Ernzerhof (*55*) exchange and correlation functionals and D3 (*56*) corrections for the van der Waals forces, including spin polarization. In the first step, the pentacene crystal structure (primitive cell, two molecules) was optimized using the FIRE algorithm (*57*), as implemented in Octopus, where a 0.16-Å̊ grid spacing was used. The optimization was stopped when no forces were higher than 0.01 eV/Å̊. Next, the laser-induced dynamics were simulated by applying a time-dependent electric field on the ground-state structure with 1.8 eV carrier frequency, a maximum amplitude of 0.1 eV/Å̊, and a Gaussian-pulse shape with τ_0_ = 6.6 fs, resulting in a pulse duration of approximately 25 fs. Ehrenfest dynamics were propagated with a time step of 0.001 fs, and the total simulation time was of 50 fs.

Molecular dynamics trajectories were obtained using the AIREBO interatomic force field (*58*) within the LAMMPS code (*59*). A 3 ×3×2 simulation cell (1152 atoms) with periodic boundary conditions is used to ensure a good description of low-frequency phonon modes. By performing a comparison of harmonic phonons, we have found that the frequencies of equivalent phonons modes with the force field and DFT can differ by up to 1 THz, likely due to the approximate nature of the empirical potential. All MDS calculations were carried out at 300 K with 0.5-fs steps. Equilibrium MDS trajectories were obtained in a two-step procedure. First, a canonical ensemble simulation using the stochastic velocity rescaling thermostat (*60*) was performed. Fifty picoseconds of continuous trajectory was obtained excluding 10 ps of initial thermalization. Next, six uncorrelated frames from thermalized NVT trajectory were extracted and used as initial configurations (positions and velocities) for consecutive NVE microcanonical simulations. The latter was performed for 15 ps. Out-of-equilibrium MDS were performed using a hotspot, nonequilibrium generalized Langevin equation thermostat (*39*) to selectively excite to 400 K a narrow range of vibrational modes in the area of 1 THz, keeping the rest of the system at a constant temperature of 300 K. Fifty picoseconds of continuous trajectory was obtained excluding 10 ps of initial thermalization. Next, six frames from hotspot simulation trajectory were extracted and used as initial configurations (positions and velocities) for consecutive NVE microcanonical simulations. These simulations were performed for 15 ps, and these data were analyzed.

## SUPPLEMENTARY MATERIALS

Supplementary material for this article is available at http://advances.sciencemag.org/cgi/content/full/7/26/eabg0869/DC1
